# Development and validation of the concise midlife crisis measure

**DOI:** 10.1038/s41598-025-23549-z

**Published:** 2025-11-13

**Authors:** Waqar Husain, Khaled Trabelsi, Angbeen Ali, Yusma Usman, Muhammad Ahmad Husain, Farrukh Ijaz, Achraf Ammar, Haitham Jahrami

**Affiliations:** 1 Department of Humanities, COMSATS University Islamabad, Islamabad Campus, Park Road, Islamabad, Pakistan; 2 Research Laboratory Education, Motricité, Sport et Santé, EM2S, LR19JS01, High Institute of Sport and Physical Education of Sfax, University of Sfax, Sfax, Tunisia; 3 Department of Movement Sciences and Sports Training, School of Sport Science, The University of Jordan, Amman, Jordan; 4 Department of Training and Movement Science, Institute of Sport Science, Johannes Gutenberg-University Mainz, Mainz, Germany; 5 Research Laboratory, Molecular Bases of Human Pathology, LR19ES13, Faculty of Medicine of Sfax, University of Sfax, Sfax, 3000 Tunisia; 6 Government Hospitals, Manama, Bahrain; 7 Department of Psychiatry, College of Medicine and Health Sciences, Arabian Gulf University, Manama, Bahrain

**Keywords:** Midlife crisis, Aging and mental health, Adult development, Middle adulthood, Measurement and assessment, Health care, Psychology, Psychology

## Abstract

Midlife crisis, characterized by emotional turbulence, identity reevaluation, and existential distress, is a psychological phenomenon often misrepresented or under-measured in both popular discourse and scientific literature. Existing global prevalence estimates are largely derived from general well-being surveys rather than standardized psychological instruments. Furthermore, there was a notable absence of concise, culturally sensitive, and psychometrically validated tools to assess midlife crisis in diverse populations. The current study aimed to develop and validate the Concise Midlife Crisis Measure (CMCM)—a brief, reliable, and conceptually grounded scale for assessing midlife crisis. The current research was conducted in two phases involving 470 participants (Mean Age = 49 years, SD = 5.29; women = 40%). The validation of the CMCM involved exploratory and confirmatory factor analyses along with convergent and divergent validity. The unidimensional CMCM, comprising 11 items (English) demonstrated excellent reliability (α = 0.954; ICC = 0.974). The model fit indices, such as CFI (0.962), TLI (0.952), RMSEA (0.089), and SRMR (0.032), showed strong validity. Convergent and divergent validity was demonstrated by the scale’s correlation (*p* < 0.001) with the Gerascophobia or Excessive Fear of Aging Scale (*r* = 0.325) and the Psychosocial Life Satisfaction Scale (*r* = -0.201), respectively. Significant inverse correlations were found between midlife crisis, age, and education. Tertile analysis revealed that approximately 32.6% of participants exhibited high levels of midlife crisis symptoms. The CMCM is a valid and reliable instrument for assessing midlife crisis in research and clinical settings.

## Introduction

Midlife crisis is a psychological phenomenon commonly experienced by individuals between the ages of 40 and 50. The phenomenon is characterized by psychological crisis brought about by events that highlight a person’s growing age, inevitable mortality, and possible lack of accomplishments in life^1,2^. It leads people to become more conscious of their mortality and make abrupt changes to pursue unfulfilled goals^3,4^.

Elliott Jaques first coined the term “midlife crisis” in his seminal 1965 paper “Death and the Midlife Crisis,” establishing it as a recognized psychological concept^2^. The theoretical foundation for understanding midlife crisis draws from Erikson’s (1950) developmental theory, particularly the generativity versus stagnation stage, which occurs during middle adulthood when individuals focus on contributing to society and guiding the next generation^5^. However, the occurrence and intensity of midlife crisis exhibit significant variation due to cultural, socioeconomic, and individual factors^6,7^. Contemporary empirical research challenges the universality of midlife crisis, indicating that many individuals experience middle age as a period of positive growth, stability, or gradual transition rather than acute crisis^8,9^. This suggests that while midlife crisis represents a valid psychological phenomenon for some individuals, it should not be considered an inevitable aspect of middle-age development.

The manifestations of midlife crisis include depressive symptoms, impulsive behaviors, and significant life changes such as career transitions, relationship changes, or major lifestyle modifications^1^. Mortality salience, or awareness of the inevitability of one’s own death, generates anxiety that triggers defense mechanisms affecting various psychological processes^4^. This heightened awareness of mortality prompts individuals to evaluate their current achievements against their earlier aspirations, often leading to existential anxiety when gaps are perceived^10^. This internal conflict intensifies when individuals experience significant failures in career advancement or relationship satisfaction, creating a sense of stagnation that contradicts their developmental needs^11^. Cultural values emphasizing individual achievement and success can further intensify midlife distress when personal accomplishments fall short of societal expectations^12^. Economic pressures also compound midlife stress, as individuals often face the dual burden of supporting aging parents while simultaneously providing for their children’s education and future needs. These financial responsibilities can strain coping resources and limit opportunities for personal fulfillment^8,13^. Additionally, major life events such as job loss, divorce, or health problems can serve as catalysts that trigger crisis episodes during this vulnerable developmental period^14^.

Personal factors significantly influence the likelihood and intensity of midlife crisis experiences. Personality traits, particularly neuroticism, predispose individuals to experience greater distress during life transitions^6^. Hormonal changes during midlife also contribute to emotional instability and crisis experiences. Research indicates that midlife experiences result from the interrelationship of biological, psychological, and sociocultural factors. For women, menopausal transitions involve declining estrogen levels that can trigger mood swings, anxiety, and feelings of loss^15^. Similarly, men experience andropause, characterized by gradually declining testosterone levels that can affect mood regulation and emotional stability^16^. These biological changes often coincide with other physical signs of aging, creating a compounded awareness of mortality and physical decline that can precipitate crisis experiences.

The earlier instruments claiming to assess midlife crises—including the Midlife Crisis Questionnaire^17^, Iranian Midlife Challenges Scale^18^, Korean Midlife Crisis Scale^19^, Chinese Midlife Crisis Scale^20,21^, and a Midlife Crisis Scale developed in India^22^—share a critical limitation: none report psychometric validation procedures. All these scales lack explicit reporting of psychometric evaluation procedures such as factor analysis and validity indices. Without rigorous validation, their reliability and construct validity remain questionable, undermining their applicability in both clinical and research contexts. The Developmental Crisis Questionnaire^23^, though methodologically sound, is not tailored specifically to the midlife period. It fails to account for the developmental specificity and phase-sensitive characteristics unique to midlife crises, treating crisis experiences as age-neutral phenomena. This limits its diagnostic precision when applied to middle-aged populations and overlooks the psychosocial transitions distinctive to midlife. Thus, the development of a psychometrically sound scale to assess midlife crisis was a pressing need in contemporary psychological research and clinical practice. As individuals between the ages of 40 and 65 increasingly encounter a range of psychosocial, existential, and physiological challenges, there is a growing recognition of midlife crisis as a distinct and complex psychological phenomenon. However, the absence of a standardized, empirically validated instrument impeded accurate assessment, early identification, and effective intervention. A well-constructed scale would not only capture the multidimensional nature of midlife crisis but also allow for the differentiation between normative developmental transitions and pathological crisis states. Moreover, such a tool would facilitate cross-cultural research, support longitudinal investigations of midlife development, and enhance clinical diagnosis and treatment planning. In an era marked by rising midlife psychological distress, shifting societal roles, and extended life expectancy, the creation of a reliable and valid measurement instrument was not just timely but essential. The current study, therefore, developed and validated the Concise Midlife Crisis Measure (CMCM). Given the growing demand for brief, time-efficient tools that respect participants’ limited attention spans and survey fatigue, the CMCM was designed to require minimal time for completion without compromising psychometric rigor.

## Methods

The study was conducted in two distinct phases. Phase one focused on the development of the CMCM and its initial validation through exploratory factor analysis (EFA). Phase two involved confirmatory factor analysis (CFA) to verify the factor structure, along with the assessment of the scale’s convergent and divergent validity.

### Development of the CMCM

The development of the CMCM was grounded in a theoretically informed and conceptually comprehensive approach. An initial pool of 20 items was constructed based on an extensive review of midlife crisis literature, with particular attention to the psychosocial, existential, and emotional challenges typically associated with this life stage. The construction of items was guided by four core themes derived from the conceptual framework of midlife crisis: identity and self-perception, life satisfaction and regret, fear of aging and mortality, and emotional and psychological distress^1,4,8,10–14^.

The first theme, identity and self-perception, included five items (I feel uncertain about who I am and what I want from life, I often question the choices I have made in my life, I feel that I am losing touch with my sense of self, I experience a strong desire to make significant changes in my life, I feel disconnected from the person I used to be) designed to assess the internal struggles individuals face in redefining their self-concept during midlife. These items capture the erosion of a coherent identity, the questioning of life choices, and a perceived disconnect from one’s earlier sense of self.

The second theme, life satisfaction and regret, comprised another five items (I am dissatisfied with my accomplishments so far, I frequently think about how my life could have been different, I feel regretful about missed opportunities in my life, I often compare myself negatively to others in my age group, I feel that I have not achieved what I wanted to by this stage in my life), addressing the retrospective evaluation of one’s achievements, unfulfilled aspirations, and social comparisons that often lead to dissatisfaction and self-critical reflections.

The third theme, fear of aging and mortality, consisted of five items targeting existential concerns and time-related urgency (I am afraid of getting older and what it means for my future, I often think about my mortality and the limited time I have left, I feel anxious about the physical changes that come with aging, I worry that my best years are behind me, I feel a sense of urgency to achieve things before it’s too late). These items were regarded sufficient to reflect the psychological impact of perceived aging, decline in vitality, and anxiety related to mortality.

The final theme, emotional and psychological distress, was represented by five items (I often feel depressed or sad about my current life situation, I experience frequent mood swings or emotional instability, I feel overwhelmed by a sense of anxiety about my future, I often feel trapped in my current life circumstances, I have difficulty finding joy or satisfaction in activities I used to enjoy), which were developed to capture the emotional turbulence characteristic of midlife crises, including symptoms of sadness, anxiety, mood instability, and emotional numbness.

Each item was crafted using clear and accessible language to facilitate respondent understanding and minimize misinterpretation, while maintaining conceptual depth and clinical relevance. The items were reviewed for face and content validity by experts in clinical psychology and lifespan development, ensuring alignment with theoretical constructs underpinning the experience of midlife crisis. This comprehensive approach to item development laid the foundation for the scale’s empirical validation through exploratory and confirmatory factor analyses.

These items were presented to a panel of five expert psychologists to determine the face validity of the CMCM. These experts had sufficient experience in psychosocial and psychometric studies. The panel confirmed that the items were valid for the construct of midlife crisis. Their agreement was also measured by interrater reliability, which reflected substantial agreement between the raters (Cohen’s weighted kappa = 0.831; Fleiss’s kappa = 0.824; Krippendorff’s alpha = 0.825). Nine items were discarded during the exploratory factor analysis because they did not have the required thresholds for validity, such as communalities less than 0.4 or cross loadings between factors above 0.2^24^. The finalized CMCM comprises 11 items (single factor) that were further validated through confirmatory factor analysis along with establishing the convergent, and divergent validity.

### Participants

The present research was conducted in two phases. A total of 470 participants (men = 280, 60%; women = 190, 40%; age = 40–60 years, M = 49, SD = 5.29; unmarried = 41, 9%; married = 429, 91%; education = primary to doctorate, M = graduation) from Rawalpindi and Islamabad, Pakistan, took part in the current study. Phase one involved 276 participants (men = 155, 56%; women = 121, 44%; age = 40–60 years, M = 48, SD = 5.09; unmarried = 26, 9%; married = 250, 91%; education = primary to doctorate, M = graduation) and phase two involved 194 participants (men = 125, 64%; women = 69, 36%; age = 40–60 years, M = 49, SD = 5.51; unmarried = 15, 8%; married = 179, 92%; education = middle to doctorate, M = graduation).

A purposive sampling technique was employed to recruit participants. The researchers individually approached participants during visits to various academic institutions, as well as government and private offices. Participation in the study was entirely voluntary, and informed consent was obtained from all participants before their involvement. The inclusion criteria required individuals to be (a) between 40 and 60 years of age, and (b) proficient in responding to questionnaires in English. The participants were approached individually by the researchers during visits to various academic institutions, government offices, and private organizations. Participation in the studies was entirely voluntary, and informed consent was verbally obtained from all individuals prior to their involvement.

The sample size for this research was determined on the basis of established guidelines for EFA and CFA where a minimum of 5 to 10 participants per item is recommended^25–27^. Our data collection exceeded this requirement and data sufficiency in both the phases was excellent (Phase 1: Kaiser-Meyer-Olkin Measure of Sampling Adequacy (KMO) = 0.950; Bartlett’s Test of Sphericity (BTS) = *p* < 0.001; Phase 2: KMO = 0.954; BTS = *p* < 0.001).

### Instruments

#### Gerascophobia or excessive fear of ageing scale (GEFAS)

The Gerascophobia or Excessive Fear of Ageing Scale (GEFAS)^28^ was used to assess the convergent validity of the Concise Midlife Crisis Measure (CMCM). The rationale for selecting this instrument lies in the conceptual overlap between midlife crisis and age-related anxieties. Midlife crisis is often accompanied by heightened awareness of aging, fear of physical and social decline, and existential concerns regarding mortality—core constructs that are central to gerascophobia. Given that fear of aging represents a salient psychological feature of midlife distress, a significant positive correlation between CMCM and GEFAS would indicate that both scales are tapping into theoretically related domains.

GEFAS consists of four items and employs a 5-point Likert scale (ranging from extremely false to extremely true). The scale has demonstrated strong psychometric properties in previous research, with high internal consistency (Cronbach’s alpha = 0.810) and significant item–total correlations (*p* < 0.001). Confirmatory factor analysis also supported its structural validity, with excellent model fit indices (Comparative Fit Index [CFI] = 0.997; Tucker–Lewis Index [TLI] = 0.982; Root Mean Square Error of Approximation [RMSEA] = 0.062; Standardized Root Mean Square Residual [SRMR] = 0.010)^28^. In the present study, the scale continued to demonstrate good reliability (Cronbach’s alpha = 0.784), supporting its use as an appropriate convergent measure for validating the CMCM.

#### Psychosocial life satisfaction scale

Life satisfaction is widely recognized as the ultimate indicator of an individual’s overall sense of fulfillment and well-being. The Psychosocial Life Satisfaction Scale^29^ was used to assess the divergent validity of the Concise Midlife Crisis Measure (CMCM). This scale was selected based on its conceptual distinction from midlife crisis; whereas midlife crisis reflects emotional turmoil, dissatisfaction, and psychological distress, life satisfaction represents a stable, positive appraisal of one’s life circumstances and psychosocial functioning. Given that individuals experiencing a midlife crisis typically report decreased satisfaction with life, a negative correlation between CMCM and life satisfaction was expected. Demonstrating such an inverse relationship would support the discriminant validity of the CMCM by confirming that it measures a construct theoretically distinct from, and inversely related to, psychosocial well-being.

The Psychosocial Life Satisfaction Scale consists of five items rated on a 7-point Likert scale ranging from *extremely unsatisfied* to *extremely satisfied*. The scale has shown excellent psychometric properties, including high internal consistency (Cronbach’s alpha = 0.872) and significant item–total correlations (*p* < 0.001). Its structural validity has also been supported through confirmatory factor analysis (CFI = 0.991; TLI = 0.981; RMSEA = 0.066; SRMR = 0.016)^29^. In the present study, the scale continued to demonstrate strong reliability (Cronbach’s alpha = 0.871), affirming its appropriateness as a divergent measure for evaluating the construct validity of the CMCM.

### Ethical considerations

This study adhered to established ethical standards, including the principles set forth in the 1964 Declaration of Helsinki and its subsequent revisions, to safeguard the rights, dignity, and well-being of all participants. Participation in the study was entirely voluntary, and informed verbal consent was obtained from all participants after a clear explanation of the study objectives. The procedure for obtaining verbal consent, including the use of a standardized script and researcher documentation at the point of data collection, was explicitly reviewed and approved by the Departmental Review Committee at COMSATS University Islamabad (Approval Code: CUI-ISB/HUM/ERC-CPA/2025-010). Participants were assured of the confidentiality of their responses, their anonymity, and their right to withdraw from the study at any point without penalty. No identifying personal information was collected, and no form of deception was employed. Additionally, participants were not exposed to any foreseeable risk or harm. All data were securely stored and utilized exclusively for scholarly purposes, in strict compliance with privacy and confidentiality protocols.

### Analysis

The collected data were entered and analyzed using the Statistical Package for the Social Sciences (SPSS, Version 26) and R for Statistical Computing (Version 4.3.2). A comprehensive data screening procedure was conducted to ensure the accuracy and integrity of the dataset. This process included checks for missing values, unengaged responses, outliers, linearity, homoscedasticity, multicollinearity, as well as assessments of skewness and kurtosis to confirm normality assumptions.

To evaluate the reliability and validity of the CMCM, both EFA and CFA were performed. EFA was conducted using the maximum likelihood extraction method with promax rotation to uncover the underlying factor structure. Key statistics examined during EFA included extraction values, KMO, BTS, and Average Variance Extracted (AVE).

CFA was subsequently employed using the maximum likelihood estimation method without rotation to validate the factor structure identified in the EFA. Model fit was evaluated through multiple indices, including the chi-square goodness-of-fit test, CFI, TLI, RMSEA, SRMR, AVE, and the Goodness-of-Fit Index (GFI). To establish the scale’s reliability, the Cronbach’s alpha and McDonald’s omega were calculated.

The psychometric properties of the MLCS were evaluated using a Polytomous Rasch Model Item Response Theory (IRT), specifically the Partial Credit Model (PCM). The scale responses were coded with the lowest category as 1, as required for polytomous modeling. Parameter estimation was performed using Marginal Maximum Likelihood Estimation (MMLE). Model fit was assessed through infit and outfit statistics, where values between 0.5 and 1.5 are considered acceptable for rating scale data. Person reliability was calculated to evaluate the internal consistency of the scale. Local independence was examined using the Q3 correlation matrix, with residual correlations above 0.20 indicating potential violation of the unidimensionality assumption. The delta-tau parameterization was used to examine threshold functioning across response categories for each item. All analyses were conducted using the snowIRT module, with the eRm R package.

Finally, Pearson’s correlation coefficient was used to examine the relationships among study variables and to assess both convergent and divergent validity of the CMCM.

## Results

### Reliability

The CMCM demonstrated strong reliability across both the EFA and CFA phases. Internal consistency was excellent, with Cronbach’s alpha coefficients of 0.962 during EFA and 0.954 during CFA (Table 1). The item–total correlations, ranging from 0.820 to 0.881 (*p* < 0.001; M = 0.586), and item–scale correlations, ranging from 0.576 to 0.854 (*p* < 0.001; M = 0.849), further confirmed the scale’s high internal consistency and discriminative capacity. Additionally, the test–retest reliability of the CMCM, assessed over a two-week interval with a subsample of participants (*n* = 30), was found to be excellent. The intra-class correlation coefficient (ICC3,1) yielded a point estimate of 0.974, with a 95% confidence interval ranging from 0.947 to 0.987, indicating high temporal stability of the instrument.

**Table 1:** Descriptive statistics, reliability, and data accuracy (*n* = 470).

Variable	Items	α	M	SD	%	Range		Skewness	Kurtosis
						Potential	Actual		
Phase 1									
Midlife crisis	11	0.962	40.742	19.146	52.91	11–77	11–77	0.168	-1.021
Phase 2									
Midlife crisis	11	0.954	43.788	18.413	56.86	11–77	11–77	0.094	-1.054
Psychosocial life satisfaction	5	0.871	22.396	6.421	63.98	5–35	5–35	-0.505	-0.470
Gerascophobia	4	0.784	17.350	5.832	61.96	4–28	4–28	-0.146	-0.127

*n* = Number of participants; α = Cronbach’s Alpha; M = Mean; SD = Standard Deviation.

Phase 1: *n* = 276; men = 155, 56%; women = 121, 44%; age = 40–60 years, M = 48, SD = 5.09; unmarried = 26, 9%; married = 250, 91%; education = primary to doctorate, M = graduation.

Phase 2: *n* = 194; men = 125, 64%; women = 69, 36%; age = 40–60 years, M = 49, SD = 5.51; unmarried = 15, 8%; married = 179, 92%; education = middle to doctorate, M = graduation.

Combined: *n* = 470; men = 280, 60%; women = 190, 40%; age = 40–60 years, M = 49, SD = 5.29; unmarried = 41, 9%; married = 429, 91%; education = primary to doctorate, M = graduation.

### Exploratory factor analysis

In the EFA (Table 2), maximum likelihood with promax rotation was implied. The sampling adequacy was notable (Table 2; *n* = 276; KMO = 0.950). The adequacy of correlations between items was highly significant (Table 2; BTS: χ² = 2875.494, *df* = 55, *p* < 0.001). Nine items *(I am afraid of getting older and what it means for my future*,* I often think about my mortality and the limited time I have left*,* I feel anxious about the physical changes that come with aging*,* I worry that my best years are behind me*,* I feel a sense of urgency to achieve things before it’s too late*,* I often feel depressed or sad about my current life situation*,* I experience frequent mood swings or emotional instability*,* I feel overwhelmed by a sense of anxiety about my future*,* I often feel trapped in my current life circumstances)* were discarded during the exploratory factor analysis because they did not meet the required thresholds for validity, such as uniqueness values above 0.60 or cross-loadings greater than 0.20^24^. The final version of the CMCM consists of 11 items, reflecting a unidimensional factor structure. The factor loadings for these items ranged from 0.780 to 0.876 (Table 2), indicating strong item–factor associations. The AVE of this single-factor solution was 69.6%, demonstrating substantial explanatory power.

**Table 2:** Exploratory factor analysis, item-total, and item-scale correlations (Phase 1; *n* = 276).

Item	Factor structure		Item-total correlations
	Loadings	Uniqueness	
1	0.876	0.232	0.820***
2	0.865	0.252	0.858***
3	0.865	0.252	0.878***
4	0.853	0.272	0.830***
5	0.849	0.280	0.843***
6	0.841	0.293	0.881***
7	0.827	0.316	0.861***
8	0.814	0.337	0.830***
9	0.805	0.352	0.863***
10	0.797	0.365	0.875***
11	0.780	0.392	0.807***

***p < 0.001.

Extraction: Maximum likelihood with Promax rotation.

Bartlett’s Test of Sphericity: χ² = 2875.494, df = 55, *p* < 0.001.

Kaiser-Meyer-Olkin Measure of Sampling Adequacy: overall value = 0.950, values for individual items ranged from 0.916 to 0.979; Total variance explained: 0.696.

Response sheet: strongly disagree (scored 1), disagree (scored 2), slightly disagree (scored 3), not sure (scored 4), slightly agree (scored 5), agree (scored 6), strongly agree (scored 7).

Items: (1) I feel uncertain about who I am and what I want from life. (2) I often question the choices I have made in my life. (3) I feel that I am losing touch with my sense of self. (4) I experience a strong desire to make significant changes in my life. (5) I feel disconnected from the person I used to be. (6) I am dissatisfied with my accomplishments so far. (7) I frequently think about how my life could have been different. (8) I feel regretful about missed opportunities in my life. (9) I often compare myself negatively to others in my age group. (10) I feel that I have not achieved what I wanted to by this stage in my life. 11. I have difficulty finding joy or satisfaction in activities I used to enjoy.

### Confirmatory factor analysis

CFA was performed on the final set of 11 items to evaluate the hypothesized unidimensional factor structure of the CMCM. The analysis employed the maximum likelihood estimation method without rotation. All factor loadings were statistically significant (*p* < 0.001), ranging from 0.703 to 0.875 (Table 3), reflecting strong associations between the items and the latent construct. The scale also exhibited excellent internal consistency, with both McDonald’s omega (ω = 0.954) and Cronbach’s alpha (α = 0.954) indicating high reliability. The CFA model demonstrated acceptable fit: χ²(44) = 111.024, *p* < 0.001, χ²/df = 2.52. Incremental fit indices indicated good fit (CFI = 0.962, TLI = 0.952, GFI = 0.957), while absolute fit indices showed SRMR = 0.032 (excellent) and RMSEA = 0.089 (90% CI = [0.068 —0.109], p-close = 0.001). Although the RMSEA value fell in the borderline range, the overall pattern of indices supports the adequacy of the unidimensional model, consistent with recommendations for multi-index model evaluation^27,30^.

**Table 3:** Confirmatory factor analysis (Phase 2; *n* = 194).

Factor	Item	Factor loadings				Residual variances			
		Estimate	SE	z	*p*	Estimate	SE	z	*p*
1	1	0.852	0.120	14.664	< 0.001	0.274	0.135	8.637	< 0.001
	2	0.799	0.117	13.300	< 0.001	0.362	0.152	9.044	< 0.001
	3	0.805	0.121	13.454	< 0.001	0.352	0.159	9.010	< 0.001
	4	0.810	0.125	13.578	< 0.001	0.344	0.168	8.990	< 0.001
	5	0.851	0.118	14.655	< 0.001	0.276	0.133	8.624	< 0.001
	6	0.875	0.113	15.334	< 0.001	0.235	0.110	8.382	< 0.001
	7	0.800	0.118	13.300	< 0.001	0.360	0.155	9.003	< 0.001
	8	0.703	0.118	11.107	< 0.001	0.505	0.187	9.398	< 0.001
	9	0.727	0.132	11.620	< 0.001	0.472	0.225	9.340	< 0.001
	10	0.867	0.119	15.124	< 0.001	0.248	0.125	8.501	< 0.001
	11	0.788	0.123	13.044	< 0.001	0.378	0.171	9.105	< 0.001

Estimation was performed using the Maximum-likelihood extraction technique with no rotation while using R (version 4.3.2) with the lavaan package.

Chi-square test: Baseline model: χ² = 1818.182, df = 55, Factor model: χ² = 111.024, df = 44, *p* < 0.001; Comparative Fit Index (CFI): 0.962; Tucker-Lewis Index (TLI): 0.952; Goodness of fit index (GFI): 0.957; Root mean square error of approximation (RMSEA): 0.089 (90% CI = [0.068 —0.109], p-close = 0.001); Standardized root mean square residual (SRMR): 0.032; Average variance extracted: 0.656, Coefficient ω = 0.954; Coefficient α = 0.954.

### Polytomous Rasch analysis

The MLCS demonstrated strong psychometric properties with a person reliability of 0.92, indicating excellent internal consistency. All 11 items showed acceptable fit statistics, with infit values ranging from 0.76 to 1.51 and outfit values from 0.69 to 1.53. Item difficulty measures ranged from − 1.89 (MLCS8, easiest to endorse) to -1.22 (MLCS1 and MLCS9, most difficult to endorse), with standard errors consistently at 0.06 (Table 4). The Q3 correlation matrix revealed mostly acceptable residual correlations, with the majority of inter-item correlations below 0.20, supporting the unidimensionality assumption. The highest residual correlation was − 0.33 between MLCS2 and MLCS9. The delta-tau parameterization showed that all items utilized the full range of response categories effectively, with threshold parameters indicating appropriate category functioning.

**Table 4:** Item statistics for the MLCS polytomous Rasch Analysis.

Item	Measure	S.E.	Infit	Outfit	Interpretation
MLCS1	− 1.23	0.06	0.85	0.78	Good fit
MLCS2	− 1.53	0.06	1.03	1.11	Good fit
MLCS3	− 1.37	0.06	1.03	1.46	Acceptable fit
MLCS4	− 1.62	0.06	1.12	1.01	Good fit
MLCS5	− 1.40	0.06	0.88	0.81	Good fit
MLCS6	− 1.28	0.06	0.76	0.69	Good fit
MLCS7	− 1.68	0.06	1.06	0.97	Good fit
MLCS8	− 1.89	0.06	1.36	1.34	Acceptable fit
MLCS9	− 1.22	0.06	1.51	1.53	Acceptable fit
MLCS10	− 1.44	0.06	0.85	0.75	Good fit
MLCS11	− 1.45	0.06	1.13	1.02	Good fit

Measure = Item difficulty in logits (negative values indicate easier endorsement); S.E. = Standard error; Infit = Information-weighted mean square statistic; Outfit = Outlier-sensitive mean square statistic. Person Reliability = 0.92. Acceptable fit criteria: 0.5 < Infit/Outfit < 1.5.

### Convergent and divergent validity

Convergent validity of the CMCM was supported by a statistically significant positive correlation with the Gerascophobia Scale (Table 5; *r* = 0.325, *p* < 0.001), indicating that higher midlife crisis scores were associated with greater fear of aging. Divergent validity was demonstrated through a significant inverse correlation with the Psychosocial Life Satisfaction Scale (Table 5; *r* = − 0.201, *p* < 0.001), suggesting that increased midlife crisis symptoms were associated with lower levels of life satisfaction.

**Table 5:** Correlation analysis.

	Midlife crisis
Psychosocial life satisfaction	− 0.201^**^
Gerascophobia	0.325^***^
Age	− 0.156^***^
Education	− 0.212^***^

**p < 0.01, ***p < 0.001.

### Midlife crisis, age, and education

Both age (*r* = − 0.156, *p* < 0.001) and education (*r* = − 0.212, *p* < 0.001) demonstrated statistically significant inverse correlations with midlife crisis scores (Table 5). These findings suggest that older individuals and those with higher levels of education reported fewer midlife crisis symptoms, highlighting the potential buffering effects of age-related experience and educational resources in navigating midlife transitions.

## Discussion

The CMCM demonstrated strong psychometric properties. It showed excellent reliability, with high internal consistency and stability over time, indicating that the instrument consistently measures the construct of midlife crisis. The exploratory factor analysis supported a clear, unidimensional structure, with well-performing items capturing the core elements of midlife crisis. The final version of the scale retained 11 items that loaded strongly on a single factor, explaining a substantial proportion of variance. The finalized 11 items of the CMCM capture the core psychological features of midlife crisis. These items collectively reflect disruptions in identity (e.g., “I feel uncertain about who I am and what I want from life”, “I feel that I am losing touch with my sense of self”), heightened self-evaluation (e.g., “I often question the choices I have made in my life”, “I feel disconnected from the person I used to be”), existential uncertainty (e.g., “I experience a strong desire to make significant changes in my life”), and emotional distress (e.g., “I have difficulty finding joy or satisfaction in activities I used to enjoy”). The scale emphasizes internal conflict stemming from dissatisfaction with life accomplishments (e.g., “I am dissatisfied with my accomplishments so far”, “I feel that I have not achieved what I wanted to by this stage in my life”), regret over missed opportunities (e.g., “I feel regretful about missed opportunities in my life”, “I frequently think about how my life could have been different”), and social comparison (e.g., “I often compare myself negatively to others in my age group”). By integrating these dimensions, the CMCM provides a theoretically grounded, multidimensional framework to assess the midlife crisis as a distinct and measurable psychological phenomenon.

The confirmatory factor analysis further validated this unidimensional structure, demonstrating a good model fit and reaffirming the scale’s structural integrity. Convergent validity was established through its meaningful association with fear of aging, suggesting that individuals experiencing a midlife crisis are also more likely to experience anxieties related to aging. Divergent validity was supported by a negative relationship with life satisfaction, indicating that higher crisis scores correspond to lower well-being.

The Polytomous Rasch analysis provides strong evidence for the psychometric quality of the MLCS. The high person reliability (0.92) indicates that the scale can reliably differentiate between individuals across the measured construct. The acceptable infit and outfit statistics for all items suggest that the items function appropriately within the Rasch framework and contribute meaningfully to the measurement of the underlying latent trait. The relatively narrow range of item difficulties (-1.89 to -1.22) suggests that all items are moderately easy to endorse, which may be appropriate depending on the construct being measured but could limit the scale’s ability to discriminate among individuals with lower levels of the trait. The Q3 correlations generally support unidimensionality, though the correlation between MLCS2 and MLCS9 (-0.33) warrants further investigation as it slightly exceeds the conventional threshold. The effective utilization of response categories across all items, as evidenced by the delta-tau parameters, supports the use of the 8-point rating scale format.

The mean percentage of midlife crisis symptoms in Phase 1 was 53%, and in Phase 2 it was 57%. Taken together, these values indicate that the overall average level of midlife crisis in our sample was approximately 55%. This proportion positions the observed prevalence in this sample notably above the 26% reported in the United States and the 20% rate in Australia^14,31^. It is comparable to the higher spectrum of the United Kingdom, where 40–60% of adults report undergoing significant midlife reevaluation^6^. Importantly, it must be noted that the prevalence figures cited from international studies are primarily derived from broad population-level surveys of well-being and subjective life satisfaction, rather than from validated psychological instruments specifically designed to assess midlife crisis. In contrast, the present study employed the CMCM, a psychometrically validated tool that captures the specific psychological manifestations of the construct, thereby offering a more precise and clinically relevant estimation. The elevated rates in this sample may reflect context-specific psychosocial stressors, including increasing socioeconomic pressure, shifting family roles, and transitional identity challenges aligned with the “sandwich generation” phenomenon^22^. In comparison, East Asian cultures such as Japan, China, and South Korea report markedly lower midlife crisis prevalence, typically under 10%, owing to cultural associations of aging with wisdom and honor^32^. While urban professionals in those societies face modern work-related midlife stress, societal frameworks mitigate identity distress through structured life course expectations^33–35^. Nevertheless, the global relevance of midlife psychological decline is supported by the U-shaped well-being trajectory, which shows lowest happiness around the mid-40s. Blanchflower (2021) and Blanchflower & Oswald (2008) report this dip at age 48 in developing countries and 47 in developed countries, reinforcing the notion that midlife distress is a widespread—though culturally filtered—phenomenon^36,37^. In this light, the 32.6% high-crisis rate in the present sample reflects a substantial and clinically significant proportion, suggesting that midlife crisis in this population is both more prevalent than in many collectivist or resource-constrained nations and on par with or exceeding individualistic societies, where personal achievement and aging anxieties dominate the psychosocial narrative of midlife.

Finally, the analysis revealed that midlife crisis symptoms tended to be lower among older individuals and those with higher educational attainment. This suggests that increased age and education serve as protective factors^1,8,16,17,19,38^, helping individuals navigate midlife transitions with greater resilience and perspective. Overall, the CMCM emerges as a concise, reliable, and valid tool for assessing midlife crisis in both research and clinical settings.

## Limitations

While the present study offers valuable insights into the assessment and prevalence of midlife crisis through the development and validation of the CMCM, some limitations must be acknowledged. First, the sample was convenience-based, drawn primarily from urban, educated, and English-speaking participants. This restricts the generalizability of the findings to broader or more diverse populations, including rural or non-English-speaking groups. Furthermore, we were unable to test measurement invariance across sex, age groups, or education levels using robust estimators. Therefore, the results should be interpreted with caution, and future research should examine the cross-group equivalence of the CMCM before making direct mean comparisons. Cultural beliefs, social norms, and developmental scripts surrounding aging, identity, and crisis vary considerably across societies. The construct of midlife crisis and its symptomatic expressions may differ significantly in other cultural or geopolitical settings. Second, even within the cultural context under study, the sample was not fully representative of the broader population. Participants were primarily drawn from urban, educated backgrounds, thereby excluding individuals from rural areas and those without formal education. This omission may have introduced a sampling bias, as educational attainment and urban living are known to influence psychological insight, self-report accuracy, and access to mental health discourse. Consequently, the experiences of midlife crisis among underrepresented groups—particularly those with limited literacy, restricted access to healthcare, or embedded in traditional community structures—remain unexplored. Future studies should strive for broader inclusivity by incorporating diverse educational, geographical, and socioeconomic subgroups. Expanding the cultural and demographic scope of research will not only enhance the ecological validity of the CMCM but also facilitate the development of culturally sensitive diagnostic and intervention frameworks for midlife psychological health.

### Implications and future directions

The findings of this study carry significant implications for both psychological research and clinical practice. First, the development and validation of the CMCM addresses a long-standing gap in the field: the absence of a psychometrically sound and time-efficient instrument specifically designed to assess midlife crisis as a distinct psychological construct. Existing global estimates of midlife crisis prevalence, often derived from broad life satisfaction or well-being surveys, lack the clinical precision afforded by standardized psychometric tools. The CMCM provides a validated alternative capable of capturing the nuanced emotional, existential, and cognitive dimensions of this life stage.

Importantly, the observation that approximately one-third of the current sample exhibited high levels of midlife crisis symptoms not only exceeds global estimates reported in the United States and Australia, but also aligns with the upper range found in the United Kingdom. This discrepancy underscores the need for context-sensitive tools and cautions against relying solely on general well-being data to assess midlife vulnerability. Furthermore, since the present data were derived using a scale specifically designed for this developmental period, they represent a more clinically meaningful estimate of midlife distress, especially in non-Western populations where cultural factors may suppress overt expression or recognition of psychological symptoms.

From a clinical standpoint, these findings call for increased screening, psychoeducation, and early intervention for individuals in the 40–65 age range, particularly those experiencing psychological distress in conjunction with health decline, caregiving burdens, or identity disorientation. Mental health professionals should consider incorporating midlife-specific assessments into routine evaluations and tailor interventions to address the existential and cultural dimensions of the crisis.

Future research should aim to validate the CMCM across diverse cultural and socioeconomic groups through cross-national and longitudinal designs. This would help delineate universal versus culturally contingent aspects of the midlife crisis and evaluate the role of protective factors such as education, social support, and generative engagement. Moreover, integration with biological markers (e.g., cortisol levels, cardiovascular indices) may advance the understanding of mind–body interplay in midlife health and crisis.

## Conclusions

This study sought to address a critical gap in psychological assessment by developing and validating the Concise Midlife Crisis Measure (CMCM), a psychometrically robust tool designed to capture the multidimensional experience of midlife crisis. Grounded in theoretical and empirical insights, the CMCM demonstrated excellent reliability, construct validity, and factorial clarity. The scale’s brevity, conceptual precision, and ease of administration make it a valuable addition to both research and clinical settings. Analyses revealed that nearly one-third of the participants experienced high levels of midlife crisis, a prevalence notably higher than international averages drawn from general well-being surveys. This highlights the need for culturally specific instruments that move beyond broad happiness indices to assess the psychological realities of midlife transitions. The findings affirm that midlife crisis is neither a universal pathology nor a cultural myth; rather, it is a contingent and context-sensitive phenomenon that warrants empirical attention and therapeutic responsiveness. By offering a validated measure, this study provides the foundation for more broad investigations into midlife development, enabling researchers and clinicians to better understand, predict, and support individuals navigating this pivotal life stage.

## Data Availability

Data associated with this paper is available at [https://osf.io/k2u9w](https:/osf.io/k2u9w).

## References

[1] Wethington, E. Expecting stress: Americans and the midlife crisis. *Motiv Emot.* **24**, 85–103. 10.1023/A:1005611230993 (2000).

[2] Jaques, E. Death and the mid-life crisis, in: is it too late? Key pap. *Psychoanal. Ageing*. 1–26. 10.4324/9780429476266-1 (2018).

[3] Lachman, M. E. Mind the gap in the middle: A call to study midlife. *Res. Hum. Dev.* **12**, 327–334. 10.1080/15427609.2015.1068048 (2015). 26848288 10.1080/15427609.2015.1068048 PMC4734389

[4] Robinson, O. C. & Wright, G. R. T. The prevalence, types and perceived outcomes of crisis episodes in early adulthood and midlife: A structured retrospective-autobiographical study, **37** 407–416. (2013). 10.1177/0165025413492464

[5] Erikson, E. H. *Childhood and Society* 1st edn (Norton & Company, 1950).

[6] Kiesow, H., Uddin, L. Q., Bernhardt, B. C., Kable, J. & Bzdok, D. Dissecting the midlife crisis: disentangling social, personality and demographic determinants in social brain anatomy. *Commun. Biol.* **4**10.1038/S42003-021-02206-X (2021). 10.1038/s42003-021-02206-x PMC8211729 34140617

[7] Alam, M. M., Ahmed, S., Dipti, R. K., Siddiquee, R. E. J. & Hawlader, M. D. H. The prevalence and associated factors of depression during pre-, peri-, and post-menopausal period among the middle-aged women of Dhaka City. *Asian J. Psychiatr*. **54**10.1016/j.ajp.2020.102312 (2020). 10.1016/j.ajp.2020.102312 32795954

[8] Freund, A. M. & Ritter, J. O. Midlife crisis: A debate. *Gerontology* **55**10.1159/000227322 (2009). 10.1159/000227322 19571526

[9] Mcfadden, J. R. & Rawson Swan, K. T. Women during midlife: is it transition or crisis? *Fam Consum. Sci. Res. J.* **40**, 313–325. 10.1111/j.1552-3934.2011.02113.x (2012).

[10] Garashchuk, S. & Pavlova, N. On the issue of life meaning orientations when living through a midlife crisis. *UniversumPsychology Educ.* **115**10.32743/unipsy.2024.115.1.16441 (2024).

[11] Jackson, M. Wilkins-Bernal-Medawar lecture Life begins at 40: the demographic and cultural roots of the midlife crisis, Notes Rec. 74 (2020) 345–364. (2019). 10.1098/rsnr.2020.0008 10.1098/rsnr.2020.0008 PMC7434712 32831409

[12] Kuther, T. L. & Burnell, K. A life span developmental perspective on psychosocial development in midlife. *Adultspan J.* **18**, 27–39. 10.1002/adsp.12067 (2019).

[13] Oluyemi, J. et al. Coping With Midlife Crisis: A Cross-Sectional Study of Ondo City, *Afr. J. Empir. Res.* **4**10.51867/ajernet4.1.24. (2023).

[14] Infurna, F. J., Staben, O. E., Lachman, M. E. & Gerstorf, D. Historical change in midlife Health, Well-Being, and despair: Cross-Cultural and socioeconomic comparisons. *Am. Psychol.* **76**, 870–887. 10.1037/amp0000817 (2021). 34914427 10.1037/amp0000817 PMC9377163

[15] Bromberger, J. T. et al. Does risk for anxiety increase during the menopausal transition? Study of women’s health across the Nation. *Menopause* **20**10.1097/gme.0b013e3182730599 (2013). 10.1097/GME.0b013e3182730599 PMC3641149 23615639

[16] Lachman, M. E., Teshale, S. & Agrigoroaei, S. Midlife as a pivotal period in the life course: balancing growth and decline at the crossroads of youth and old age. *Int. J. Behav. Dev.* **39**, 20–31. 10.1177/0165025414533223 (2015). 25580043 10.1177/0165025414533223 PMC4286887

[17] Oles, P. K. Towards a psychological model of midlife crisis. *Psychol. Rep.* **84**10.2466/pr0.1999.84.3c.1059 (1999). 10.2466/pr0.1999.84.3c.1059 10477923

[18] Roozbehani, A. Construction and validation of Iranian midlife challenges scale. *J. Psychol. Clin. Psychiatry*. **9**10.15406/jpcpy.2018.09.00602 (2018).

[19] Chang, H. K. Influencing factors on mid-life crisis. *Korean J. Adult Nurs.* **30**10.7475/kjan.2018.30.1.98 (2018).

[20] Shek, D. T. L. Midlife crisis in Chinese men and women. *J. Psychol. Interdiscip Appl.* **130**10.1080/00223980.1996.9914993 (1996). 10.1080/00223980.1996.9914993 8618211

[21] Shek, D. T. L. A scale for the assessment of midlife crisis in Chinese people. *Psychol. Int. J. Psychol. Orient.* **38** (1995).

[22] Vijayalakshmi, S. Mid-life crises among the sandwich generation people. *Indian J. Ment Heal*. **4**10.30877/ijmh.4.4.2017.380-384 (2017).

[23] Petrov, N., Robinson, O. C. & Arnett, J. J. The developmental crisis questionnaire (DCQ-12): psychometric development and validation. *J. Adult Dev.* **29**10.1007/s10804-022-09403-w (2022).

[24] Osborne, J. W., Costello, A. B. & Kellow, J. T. Best Practices in Exploratory Factor Analysis, in: Best Pract. Quant. Methods, SAGE Publications, Inc., 2455 Teller Road, Thousand Oaks California 91320 United States of America, : pp. 86–99. (2008).

[25] Comrey, A. L. & Lee, H. B. *A First Course in Factor Analysis* 2nd edn (Taylor & Francis Group, 1992). 10.4324/9781315827506

[26] Tabachnick, B. G., Fidell, L. S. & Ullman, J. B. *Using Multivariate Statistics* 6th edn (Pearson, 2013).

[27] Kline, R. B. *Principles and Practice of Structural Equation Modeling* (Guilford, 2023).

[28] Husain, W. et al. Gerascophobia or excessive fear of aging scale (GEFAS): Development, validation, and exploration of psychometric properties of a brief instrument using classical testing theory and item response theory. *Arch. Gerontol. Geriatr.* 128. 10.1016/j.archger.2024.105599 (2025). 10.1016/j.archger.2024.105599 39168076

[29] Husain, M. A. & University Islamabad, C. O. M. S. A. T. S. Validation of Psychosocial Life Satisfaction Scale, Pakistan., (2024). 10.13140/RG.2.2.29649.44648

[30] Hu, L. T. & Bentler, P. M. Cutoff criteria for fit indexes in covariance structure analysis: conventional criteria versus new alternatives. *Struct. Equ Model.* **6**, 1–55. 10.1080/10705519909540118 (1999).

[31] Melbourne Institute, H. I. L. D. A. & Survey Household, Income and Labour Dynamics in Australia, Melbourne, (2021). https://melbourneinstitute.unimelb.edu.au/hilda

[32] Perrig-Chiello, P. S.P.-J. of adult development, undefined Biographical transitions from a midlife perspective, Springer 12 (2005) 169–181. (2005). 10.1007/S10804-005-7085-X

[33] Hinata, A. et al. Education, household income, and depressive symptoms in middle-aged and older Japanese adults. *BMC Public. Health*. **21**, 2120. 10.1186/S12889-021-12168-8 (2021). 34794416 10.1186/s12889-021-12168-8 PMC8600755

[34] Karasawa, M. et al. Cultural perspectives on aging and well-being: A comparison of Japan and the united States. *Int. J. Aging Hum. Dev.* **73**, 73–98. 10.2190/AG.73.1.D (2011). 21922800 10.2190/AG.73.1.d PMC3183740

[35] Matewe, B. Midlife crisis’ and the scramble for fresh personal purposes amongst middle-aged men. *Glob J. Psychol. Res. New. Trends Issues*. **13**, 117–125. 10.18844/gjpr.v13i2.8944 (2023).

[36] Blanchflower, D. G. Is happiness U-shaped everywhere? Age and subjective well-being in 145 countries. *J. Popul. Econ.* **34**10.1007/s00148-020-00797-z (2021). 10.1007/s00148-020-00797-z PMC7480662 32929308

[37] Blanchflower, D. G. & Oswald, A. J. Is well-being U-shaped over the life cycle? *Soc. Sci. Med.* **66**10.1016/j.socscimed.2008.01.030 (2008). 10.1016/j.socscimed.2008.01.030 18316146

[38] Ryff, C. D. & Singer, B. H. Know thyself and become what you are: A Eudaimonic approach to psychological well-being. *J. Happiness Stud.* **9**, 13–39. 10.1007/S10902-006-9019-0 (2008).

